# Peer-mentoring for first-time mothers from areas of socio-economic disadvantage: A qualitative study within a randomised controlled trial

**DOI:** 10.1186/1472-6963-8-46

**Published:** 2008-02-27

**Authors:** Christine A Murphy, Margaret E Cupples, Andrew Percy, Henry L Halliday, Moira C Stewart

**Affiliations:** 1 Royal Group of Hospitals, Belfast, Northern Ireland; 2 Division of Public Health Medicine and Primary Care, Queen's University, Belfast, Northern Ireland; 3 Royal Group of Hospitals, Belfast, Northern Ireland; 4 Regional Neonatal Unit, Royal Maternity Hospital and Department of Child Health, Queen's University Belfast, Northern Ireland; 5 Department of Child Health, Queen's University, Belfast, Northern Ireland

## Abstract

**Background:**

Non-professional involvement in delivering health and social care support in areas of socio-economic deprivation is considered important in attempting to reduce health inequalities. However, trials of peer mentoring programmes have yielded inconsistent evidence of benefit: difficulties in implementation have contributed to uncertainty regarding their efficacy. We aimed to explore difficulties encountered in conducting a randomised controlled trial of a peer-mentoring programme for first-time mothers in socially disadvantaged areas, in order to provide information relevant to future research and practice. This paper describes the experiences of lay-workers, women and health professionals involved in the trial.

**Methods:**

Thematic analysis of semi-structured interviews with women (n = 11) who were offered peer mentor support, lay-workers (n = 11) who provided mentoring and midwives (n = 2) who supervised the programme, which provided support, from first hospital antenatal visit to one year postnatal. Planned frequency of contact was two-weekly (telephone or home visit) but was tailored to individuals' needs.

**Results:**

Despite lay-workers living in the same locality, they experienced difficulty initiating contact with women and this affected their morale adversely. Despite researchers' attempts to ensure that the role of the mentor was understood clearly it appeared that this was not achieved for all participants. Mentors attempted to develop peer-mentor relationships by offering friendship and sharing personal experiences, which was appreciated by women. Mentors reported difficulties developing relationships with those who lacked interest in the programme. External influences, including family and friends, could prevent or facilitate mentoring. Time constraints in reconciling flexible mentoring arrangements with demands of other commitments posed major personal difficulties for lay-workers.

**Conclusion:**

Difficulties in initiating contact, developing peer-mentor relationships and time constraints pose challenges to delivering lay-worker peer support. In developing such programmes, awareness of potential difficulties and of how professional support may help resolve these should improve uptake and optimise evaluation of their effectiveness.

Trial Registration Number: ISRCTN55055030

## Background

Evidence of the adverse effects of socio-economic deprivation on the health of young mothers and their infants is well established [1]. Providing appropriate support to address their health and social needs and to redress growing inequalities in health is important [2].

The individual needs of different mothers have been recognized [3] and various schemes have been developed to provide support on a one-to-one basis [4-6]. Many such programmes involve lay support workers, working with supervision from health professionals. Non-professional involvement is thought to be especially important in providing health care for 'hard to reach' groups [7], and has been credited with extending wider benefits to their local communities [7,8]. Mothers who have availed of lay-worker support rate it highly [4,9-14]. However, in controlled trials positive reports by users have not translated consistently into measurable benefit for intervention groups. For example, infants in the Community Mothers Programme in Dublin [4] had improved outcomes in relation to immunisation status and diet but similar studies in the UK [9,10,13,15] did not confirm these findings.

Possible explanations for the apparently contradictory evidence include difficulties in measuring maternal and infant well-being [13], and programme difficulties such as poor uptake of the intervention [10,11,14,15] and high staff turnover among lay-workers [9,15-17]. Whilst reasons for difficulties in their implementation, such as lack of clarity of the role of the lay worker have been reported, these have not been fully explored and their exploration has relevance to resolution of the uncertainty regarding efficacy of programmes.

We conducted a randomized controlled trial (RCT) of peer mentoring for women, living in areas of socio-economic disadvantage, who became first-time mothers. Our objective was to determine whether peer mentoring support during pregnancy and the first year of infant life could improve child health and maternal outcomes. The primary outcome measures recorded at one year after birth were the Bayley Scales of Infant Development (assessing mental, motor and behavioural performance), and the SF-36 (assessing maternal physical and mental health). Secondary outcomes included assessment of fetal behaviour, birth gestation, infant feeding, growth at one year, immunization uptake, parental efficacy, maternal diet, smoking, alcohol and drug use, and use of health and social services.

Within the context of the randomized trial we used qualitative methods to explore the difficulties experienced by lay-workers, women and health professionals involved in the peer-mentoring programme. The qualitative findings are reported in this paper.

## Methods

### Study setting

This study took place in socially deprived areas of Belfast. Women, aged 16–30 years, living in areas of high socio-economic deprivation (identified by postcode), who had no previous pregnancy and required no ongoing healthcare for other conditions were, with ethical committee approval (Application No 124/03, Central Office for Research Ethics Committees), identified in hospital antenatal clinics (Nov 2003 – Feb 2005) and invited to participate in a RCT of peer mentoring. Before agreeing to take part they were told that their participation might involve receiving two-weekly visits from a mentor, a lay person (not a professional health or social care worker) who would be a mother who lived in the same locality as they did and who had at least one young child, and that the visits would be arranged to suit them, would normally take place in their own home and would continue throughout pregnancy and for one year after the child was born. During the visits they would be offered advice about their own and their baby's healthcare and help in accessing professional health and social care services as required. If they agreed to participate their mentor would telephone them as soon as possible after their hospital appointment. Participation would also involve filling in questionnaires about their health, lifestyle and parenting experiences, having an additional ultrasound scan to observe the baby's behaviour at 29 weeks of pregnancy, and allowing researchers to access their infant's medical records and perform a physical examination of the child at one year. This information was given to them in writing and they were invited to ask questions to ensure their understanding. Their informed consent was obtained by a research midwife prior to their inclusion in the study.

### Mentor selection and training

Mentors were selected following response to advertisements in local press and community centres. They were of similar age to the participants, lived in the same localities and had at least one child under 10 years of age. They were paid travelling and telephone expenses and £6 for every hour spent in association with the programme, including participation in training sessions. At the start of the trial mentors were given, in each of the first three weeks, one formal two hour training session at which the programme and the role of the mentor were explained. Mentors were advised that their role was to identify health and social care needs of the women, to ensure awareness of health promotion information and to provide non-professional social support. The limitations of their role were emphasized and they were told how to refer women to appropriate statutory or voluntary services if they had specific queries regarding their health or social care. If there was any doubt about appropriate action, mentors were encouraged to contact the midwives directly for advice.

**Figure 1:**
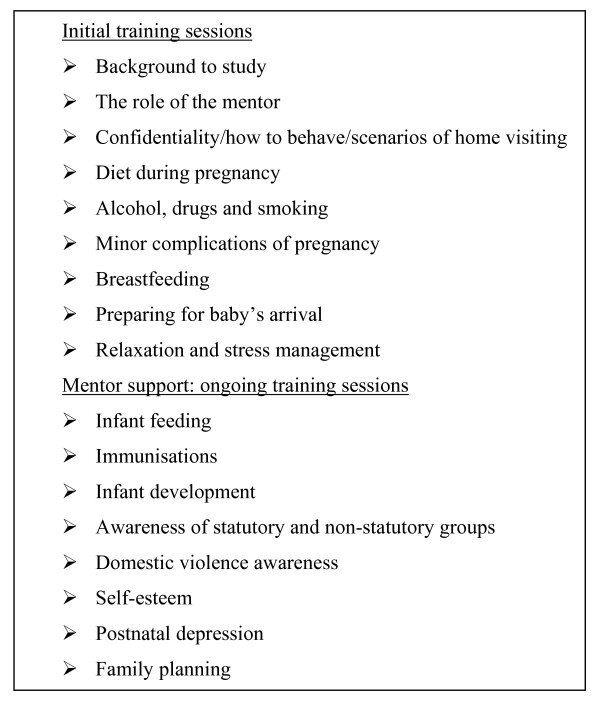
Programme for training of mentors.

The RCT project manager (CM) organized the training in collaboration with the two research midwives. Information relating to pregnancy, postnatal self-care, infant care, communication skills and awareness of safety issues in conducting home visits was delivered by health and social care professionals (Figure 1). In addition to being given written handouts highlighting the major issues, mentors were given written materials (such as leaflets produced by the Health Promotion Agency, Northern Ireland) which they could share with the women whom they mentored. The mentors had direct telephone access to the midwives from 08:00 to 18:00 on weekdays and either by leaving a message for a return call or by the project manager's mobile telephone at other times. Midwives contacted mentors at approximately two-weekly intervals if they had not initiated contact during that time.

Further training was provided as deemed appropriate by the midwives, subsequent to the mentors encountering difficulties or questions. Mentor group meetings took place every six to eight weeks, at mutually convenient times and settings, when peer support was available through sharing of experiences. The research midwives attended these meetings but gave the mentors opportunity to talk together informally. Following mentors resigning from the programme, the midwives trained replacement mentors on a one-to-one basis, or in small groups if possible, placing initial focus on topics most pertinent to the stage of pregnancy of the women assigned to them. Further training took place through the ongoing mentor support meetings. Each mentor self-completed a training log throughout the programme.

Mentors contacted the women assigned to them as soon as possible after their clinic visit, to provide support, through home visits by telephone, tailored to individual needs.

### Data collection

Purposive samples were selected for invitation to participate in this qualitative study. Mentors were selected to include a range of age, locality, work experience, family composition and mentoring experience. Women were selected to include different ages, localities and mentor experiences. With their consent, semi-structured one-to-one interviews were conducted with mentors, women and research midwives, beginning ('early interviews') nine months after the start of the trial, so that the process of mentoring had become established. Interviews with six mentors were conducted and analysed before interviewing women and midwives. After an initial analysis further interviews ('later interviews') with mentors, women and midwives began approximately one year later. Selected mentors and women were each interviewed only once in order to maximise sample diversity; both research midwives were interviewed at both stages of the study.

**Figure 2:**
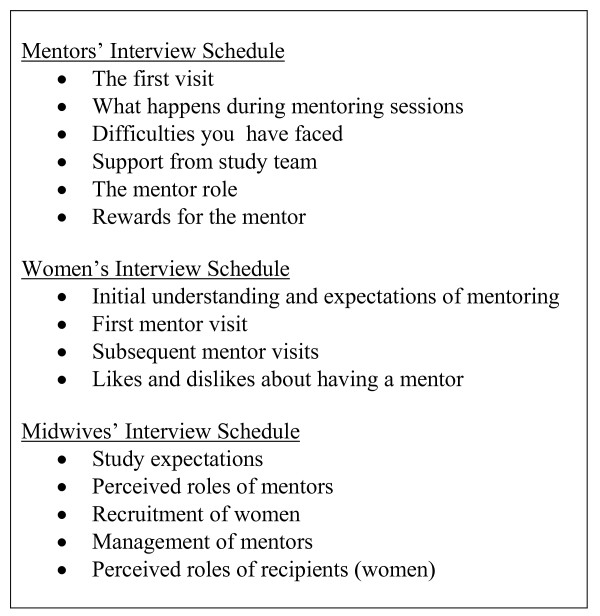
Interview topics and schedules for mentors, women and midwives.

Questions were based on the findings of previous trials of lay worker support for mothers and on the project manager's observations of the RCT implementation (Figure 2). Mentors and women were interviewed at a venue of their choice (home or hospital) by CM. Another researcher (AP), not involved in the RCT management, interviewed the midwives in a hospital room. Interviews were tape-recorded with the interviewees' consent and the interviewer recorded observations.

Themes identified in earlier interviews were explored further in later interviews.

To attempt to confirm the validity of the qualitative findings further data were collected by administering a postal questionnaire, containing questions based on the themes identified, to all mentors involved in the RCT, including those who resigned.

### Data Analysis

Interviews were transcribed verbatim into Microsoft Word and analysed by two researchers independently [18] (CM, AP) using principles of grounded theory [19] to develop a coding framework. The emerging framework was reviewed by researchers (CM, AP, MC) to condense codes into categories and identify themes. These explanations for the data were then compared with the original transcripts to ensure consistency and identify any cases where views expressed differed from those described in relation to each theme. Iterative analysis allowed themes to be explored more fully in later interviews. Observations recorded at the interviews were examined to ensure that the transcripts provided a comprehensive account of the issues involved [20]. The researchers (CM, AP, MC) reviewed the transcripts to confirm that data saturation was achieved.

## Results

**Figure 3:**
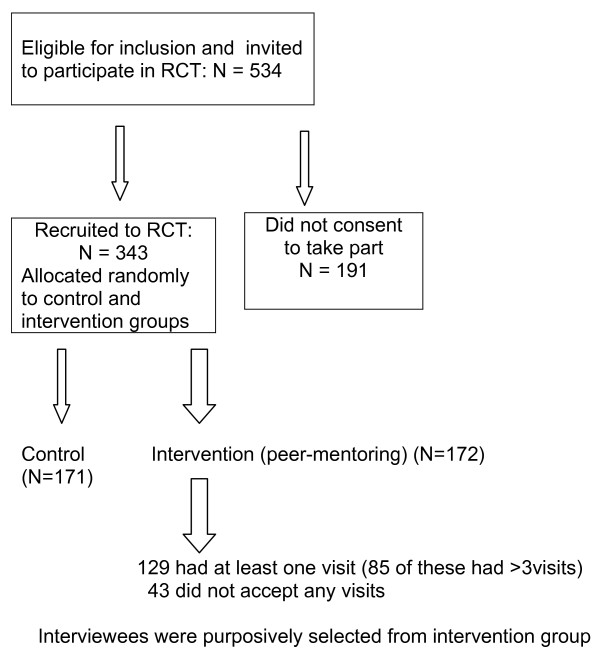
Flow chart showing progress through randomised controlled trial to qualitative study.

Of 534 eligible women, 343 (64%) agreed to participate in the RCT; 172 were allocated to receive peer-mentoring (Figure 3). Of these, 129 (75%) received at least one visit from a mentor; 85 (49%) received three or more visits. Of the 32 mentors involved in the RCT, 11 were invited to participate and all agreed. Twelve women were invited to participate in interviews and 11 agreed. Table 1 shows some characteristics of the interviewees.

**Table 1:** Characteristics of women and mentors interviewed

**Women interviewed**	**Age (years)**	**Number of visits by Mentor**		**'Early' (E)/'later'(L) interview**
1	16	6		E
2	29	6		E
3	23	4		E
4	17	1		E
5	19	3		E
6	19	5		E
7	30	2		E
8	20	0		E
9	19	16		L
10	25	14		L
11	22	>20		L

**Mentors interviewed**	**Age (years)**	**Number of children**	**Age of youngest (years)**	**'Early' (E)/'later'(L) interview**

1*	23	2	2	E
2	36	2	4	E
3	26	1	7	E
4	33	4	2	E
5*	34	2	9	E
6	33	4	2	E
7	25	1	2.5	L
8	27	2	0.75	L
9*	29	1	1.3	L
10	25	2	1	L
11	31	2	2	L

* Women mentored included two from ethnic minority backgrounds

Three main themes relating to difficulties experienced were identified:

(i) Initiating peer-mentoring

(ii) Developing the peer-mentor relationship

(iii) External influences on mentoring

### Initiating peer-mentoring

#### Initial peer-mentor contact

Initial mentor contact was never achieved for 25% of the women allocated to receive mentoring. Mentors reported difficulties, including incorrect or unanswered telephone numbers, no response to messages, postponement of arranged visits and women not being at home for arranged visits. In early interviews mentors reported adopting a sympathetic approach, trying to establish rapport through telephone conversations and re-arranging numerous appointments.

*'Someone said that's terrible you arrange a visit with somebody, you get childminders organised and they are not in. But then it came back to me..... you have to be a bit wise to what the other person's going through....put yourself back in their situation' *(Mentor 1, Early interview)

Repeated unsuccessful attempts at contact appeared to affect some mentors' personal morale.

*' you were leaving message after message and it wasn't coming back and it was a bit disheartening like *(Mentor 2, Early interview)

In response to being asked about their role in management of mentors, the midwives reported that they recognised a need to support mentors in initiating contact in order to try to encourage mentors to stay in the programme.

*' If they *(mentors)*were having any difficulty getting in touch with the mums we would have taken that off them and we would have tried to get in touch with them or we would have spoken to them then whenever they came up to the clinic' *(Midwife 1, Early interview)

Interestingly, later interviews did not reveal such difficulties in initiating contact but suggested that mentors then had a less sympathetic approach, and reported failed contacts to the midwives more readily.

*'Two mothers didn't turn up for their visit, but then I never got to see them, cause I phoned R (research midwife) and she spoke to them and it turned out that they didn't want a mentor' *(Mentor 7, Later interview)

#### Understanding of the mentor's role

Women appeared to have a poor understanding of the RCT and of the role of the mentor. This was confirmed by mentors who reported that some women perceived them to be health or social care professionals.

*'I didn't know about the visits and all. I thought it was just questionnaires and stuff at the hospital. I really didn't know what one *(a mentor) *was and all' *(Woman 1, Early interview)

*'One of the girls I went to thought I was a social worker and she couldn't understand why I was there' *(Mentor 4, Early interview)

Whilst none of the women had positive expectations about what mentoring could achieve for them, all who were interviewed reported positive experiences of mentoring. One woman's expectations were clearly negative, in contrast to her positive experience.

*'I thought she was actually going to be a nuisance... but she was great' *(Woman 1, Early interview).

Midwives perceived that women's interest in the RCT lay in the opportunity to avail of some outcome assessments, such as the 29 week fetal behaviour scan, rather than in receiving mentoring. They also suggested that some mentors failed to appreciate that mentoring involved more than providing superficial social contact.

*'A lot of the girls*(women)*maybe just come into the study, maybe not for the mentorship part of it, maybe only they like the idea of the baby outcome, the scan, they haven't really thought about the mentorship part' *(Midwife 1, Early interview)

*'...I really don't think they *(mentors) *realised what the job entailed, it just wasn't going in for a cup of tea and a chat about your new baby that they actually had to introduce the diet, domestic violence, feeding etc. I am not sure, some of them just did a couple of visits and then said 'no thanks'.' *(Midwife 1, Early interview)

### Developing the peer-mentor relationship

Having overcome the barrier of initial contact, several mentors reported that they began establishing a relationship by offering friendship and talking about their personal experiences of pregnancy and motherhood. They hoped thereby to gain trust and facilitate discussion of sensitive issues in subsequent visits.

*'I always try at the first (visit) to talk about everything. Not just pregnancy. So I would probably take the lead and tell them about my kids, what age they are...' *(Mentor 4, Early interview)

*'The second time was far more open you could talk about anything. The third time you were able to talk abut breast-feeding and more personal things... how fat they feel and how big they are getting, whereas before they wouldn't have mentioned how fat they felt or how tired' *(Mentor 5, Early interview)

Women valued the social support offered, the time mentors spent with them and the shared personal information and experiences.

*'She .... actually spent time. There were times she was maybe here for an hour to 1 1/2 hours and she wasn't in a rush or anything, she was good' *(Woman 3, Later interview)

*'... some midwives say wash the baby's clothes separately and some say to wash them with your own clothes. I would ask her things, like what did you do.....' *(Woman 3, Later interview)

Midwives reported perceptions that successful peer-mentorship involved friendship and a high level of practical support.

*'collected her from hospital when she had her baby .....Like another friend ......someone who she could rely on who wouldn't let her down' *(Midwife 2, Early interview)

Mentors reported difficulties in providing any support in situations where a friendship bond did not develop and when there was disinterest or lack of perceived need. Some reported failure in trying to achieve satisfactory communications with the women.

*'I said 'what about the parentcraft?' *(group classes, giving information about pregnancy, labour and childcare) *to prepare for and she said 'I don't think I'll go to that'. There was no relationship there to build on' *(Mentor 4, Early interview)

*'You were going in and coming out really frustrated – non-communication, didn't even acknowledge you, just staring out the window....., that was a real difficulty' *(Mentor 6, Early interview)

Midwives reported that in some situations, particularly where there was extended family support, mentors would find difficulty establishing relationships and in achieving positive change because of the women receiving advice from other sources.

*'mother or older sister has just had a baby or grandmothers are sometimes still involved, so they would maybe hang on their ideas and their words as opposed to the mentor' *(Midwife 1, Early interview)

### External influences on peer mentoring

#### Ethnicity

Mentors reported communication difficulties with women of different ethnic backgrounds; these women could speak English but at times they appeared to lack understanding. This was sometimes attributed to a failure to understand local sayings, 'slang' words or culture and was perceived to be a barrier in developing the peer-mentor relationship.

*'Sometimes when she would say 'yes' I wasn't sure that she knew what I meant' *(Mentor 8, Later interview)

*'I would have made a joke out of it. But you can't do that with the culture difference' *(Mentor 3, Early interview)

Mentors reported discovering important cultural differences for which they were unprepared and feared causing offence.

*'I didn't understand the reaction I got from her. She then told me, she is from XXX, and there they hold their babies on the potty every day to go to the toilet properly....... it is something you don't know' *(Mentor 8, Later interview)

In the context of having had such experiences, mentors gave these women information through pre-set agendas rather than by responding to any identified need. Despite this however mentors felt that their visits to them were worthwhile as they appeared to have little local social support. This view was supported by observations of the relatively high rate of mentor visits accepted by the women with minority ethnic backgrounds in the RCT: of the 10 who were assigned a mentor, only one did not avail of any visits.

#### Involvement of others

Mentors reported difficulties when other people were present at mentoring visits; some interrupted exchange of information, others wanted to discuss their personal concerns.

*'... when I put a question to her – he answered. She didn't.' *(Mentor 6, Early interview)

*' One day I was there for an hour and forty-five minutes talking just with the mother *(woman's mother about her depression)*' *(Mentor 7, Later interview)

Communications between women and mentors were also disrupted when others present did not participate in conversation, despite invitations to do so.

*'He always sat and watched the TV and I always felt rushed when he was there, whereas if she was on her own she could talk more...'*(Mentor 4, Early interview)

However, mentors also reported positive experiences of support from relatives and partners.

*'...we would sit and talk away about her pregnancy and how she has been doing and all and if she's not eating probably the granny would actually tell me' *(Mentor 1, Early interview)

Women reported that one-to-one visits gave more opportunity to discuss personal issues.

*'To be honest I liked it better with just me and XX............, sometimes I would probably have asked her questions without my mummy being there'* (Woman 1, Early interview)

Mentors were also aware of the potential negative influences of others, outside of the visits, either by contradicting information or by interfering with women's continued participation in the programme.

'*XX was interested in breast feeding so I gave her lots of information about that. She was keen but her boyfriend's relatives were a bit negative about it' *(Mentor 7, Later interview)

*' ..that was the last I seen of her cause her partner always answered the phone and saying she's not here...'*. (Mentor 5, Early interview)

In contrast, some women reported how others outside the mentoring programme could benefit from it.

*'Me and my sister were pregnant at the same time. Some of the stuff that XX gave me I shared it with my sister too. I actually still have it as well.' *(Woman 3, Later interview)

#### Time constraints

Several mentors reported struggling to fit the mentoring around their other work and family commitments even though the number of hours per month for mentoring was small (from 1 to 11 hours). Mentors identified that difficulty contacting women and finding mutually convenient times added to their workload.

*'.. it is not like you can just say one afternoon a week-everyone wants to meet at different times and then when they don't turn up you have wasted a whole afternoon. So I don't know if it was a long term thing if I could really do it. I love seeing the mums and babies but it has been quite hard time wise' *(Mentor 7, Later interview)

*' ... there is a lot more work *(than just the meetings)*doing it, a lot more trying to phone people and not getting them in ......and it was trying to fit my time in as well as them' *(Mentor 2, Early interview)

The questionnaire confirmed that time was considered an issue for all but two of the mentors who completed the questionnaire (n = 13); nine of the 22 who resigned during the programme cited time constraints as the reason for their resignation.

## Discussion

Our findings suggest that difficulties in delivering a peer-mentoring programme to women who become first-time mothers living in socio-economically deprived areas relate to three main themes – initiating contact, developing the peer-mentor relationship and external influences, particularly of other people and time constraints. Retention of mentors in the programme reflected their ability to overcome difficulties encountered in establishing face-to-face contact, understanding the concept of mentoring, building relationships through offering practical friendship, sharing problems with professionals and being flexible in time availability. The study adds to previous reports of how individuals value support provided by lay-workers [4,9-14] and highlights the unique way in which peer mentors can provide social support and health information in the context of personal experience. It provides information which may help optimise the development, implementation and evaluation of future interventions.

### Uptake of mentoring

Despite positive reports from users, poor uptake of lay-worker support programmes has been reported [9,11,14,15]. Interventions provided by health professionals show better uptake rates [3,15] even where the intervention programme has been identical [21]. Previously reported reasons for non-usage of community schemes include failure of intended recipients to open doors to lay-workers [3,9-11,13], failure to make contact because of staffing problems [8,9] and non-commitment by health professionals in supporting these [7,9,11]. We sought to take account of these difficulties in our selection and training of mentors; the research midwives were totally committed to the project. However, our findings suggest that the purpose and planned outcomes of our programme could have been explained more clearly. Participants' reports provide insight into women's low expectations of personal gain from the programme and some mentors' understanding of 'mentoring'. These perceptions may be helpful in future mentor training and when inviting women to take part in such programmes. Our findings support Wiggins et al's suggestion that schemes should provide a clear outline of what mentoring could provide and what outcomes may be achieved [15].

In a research context it is important to try to minimise the contribution of poor understanding of the mentor's role to poor uptake and usage of programmes and thus maximise the chances of demonstrating significant differences between intervention and control groups. There also are real consequences for communities, with possible increasing health inequalities, if such programmes are associated with significant health benefit for mothers and infants but fail to be used by those in most need.

### Provision of mentoring

Successful mentoring provision appears to be associated with good peer-mentor relationships, which clearly requires retention of mentors within a programme. Our findings indicate that difficulties in making initial contact have adverse effects on mentors' morale, cause problems in their time management and contribute to staff turnover. It is possible to plan supporting mentors who are office based and work specified hours but this would reduce time flexibility and weaken their role as a lay-worker based within the community. Our reports of potential negative and positive influences of other people and the importance of awareness of different cultural practices, which have previously been identified [15], should further inform future lay-worker training and support within peer-mentoring programmes.

In our earlier interviews mentors reported a sympathetic approach, to difficulties in contacting women, which was not identified in later interviews. Whilst it may be suggested that the personalities of mentors involved in 'later interviews' differed from those involved in the 'early interviews', we do not consider that this was so. Observations by the project manager confirmed that the research midwives became more persistent in their questioning of mentors' progress over the course of the trial as they became more aware of the difficulties and the consequent implications for failing to meet trial recruitment targets and adverse effects on mentor morale. They felt mentors were reluctant to admit failure initially but became less so as, in ongoing support meetings, shared experiences revealed that others had similar difficulties.

### Limitations and strengths

Findings from this qualitative study cannot be generalised but are in keeping with previous reports of lay-worker schemes in other settings and add depth of information to those. It must be acknowledged that we did not interview any women from different ethnic backgrounds. The number of these who took part in our RCT was small as there was a low prevalence of ethnic diversity in the target population in Belfast at the time of the study. Of the 10 women from different ethnic backgrounds who participated in the intervention arm of the RCT we had hoped to interview at least one at the later stage of the study but none was available – some having returned to their country of origin and all having moved out of the area.

Whilst we identified that good peer-mentor relationships are important in ensuring uptake of programmes, we did not attempt to assess the strength of friendship bonds within relationships. Also, because we wished to include as wide a range of experiences as possible, we did not re-interview the same women or mentors, so that we cannot assess changes within individuals' experiences or perceptions over time. We did not attempt to validate reports by comparing interviews of women and their assigned mentors because we did not wish participants to perceive any possible barriers to frank disclosure of their experiences. However, the transcripts were reviewed by three researchers who sought to identify any statements which were inconsistent with the themes reported and who confirmed that data saturation was achieved.

Themes emerging from early interviews were explored and confirmed in later interviews. Perceptions of the adverse impact of initial contact difficulties on mentor morale and time management were confirmed by the questionnaire responses. These, alongside mis-perceptions of what mentoring involved, difficulties in establishing relationships with women assigned to them and alternative employment opportunities, usually with less need for flexibility in working hours, were confirmed as contributing reasons for resignation.

### Implications for practice

This study highlights some of the difficulties encountered in providing a peer-mentoring programme for first-time mothers living in areas of socio-economic disadvantage. It also reveals some practical aspects of mentoring which were appreciated by women. The findings inform planning and delivery of future programmes which involve a complex health service intervention [22] such as peer-mentoring. Various components may contribute to the effectiveness of such programmes, including details of the intervention itself (mentor training, information given to women, strength of peer-mentor relationships, frequency and duration of contact), the women (interest in health or lifestyle change, personal experiences, other 'advisors'), mentors (level of skill, availability and flexibility of time, own experiences) and health professionals (willingness to allow mentors autonomy, identification of mentor needs, availability) and the social environment in which the mentoring takes place, both in the immediate family setting and the wider community. The design and evaluation of such interventions present challenges [23] and require careful consideration of these possible interactions. Understanding an intervention makes an important contribution to development of its evaluation and the provision of information which is meaningful for translation into health service policy and practice.

## Conclusion

Exploration of experiences within a research trial of a peer-mentoring programme for first-time mothers in a disadvantaged area has revealed how difficult it is to communicate clearly what the role of a mentor involves to both lay-workers and potential recipients of such a programme. It is important to outline what the scheme will mean for participants and clarify their understanding and expectations. There are difficulties in defining limitations for the outworking in practice of the 'social capital' invested within the concept of friendship/social support for women and their mentors. It is difficult also to specify the expected personal gain for participants or predict the time and effort required by mentors in achieving effective delivery of a programme to individual women.

The challenges for mentors in making contact with intended programme recipients should not be underestimated. Potential mentors require communication and time management skills and a level of self-confidence which enables them to deal with difficult home-visiting situations and to share problems with health professionals. Clarification of these details is relevant to improving uptake of such schemes and retention of lay-workers within them. This knowledge should be used in further evaluation of the effectiveness and cost-effectiveness of lay-worker schemes for improving health outcomes for mothers and their children living in areas of socio-economic deprivation.

## Competing interests

The author(s) declare that they have no competing interests.

## Authors' contributions

CM interviewed the mothers and mentors, analysed data and wrote the initial draft of the paper. MC was involved in the study design, data analysis and in writing and revising the manuscript. AP was involved in the study design, interviewed the midwives, analysed data, and contributed to revision of the manuscript. HH is the lead investigator for the trial within which this study took place and was involved in reviewing the data and revising the manuscript. MS was involved in the study design, mentor training and revising the manuscript. All authors approved the final version of the manuscript.

## Pre-publication history

The pre-publication history for this paper can be accessed here:


